# Substituting the catalytic proline of 4-oxalocrotonate tautomerase with non-canonical analogues reveals a finely tuned catalytic system

**DOI:** 10.1038/s41598-019-39484-9

**Published:** 2019-02-25

**Authors:** Michael S. Lukesch, Tea Pavkov-Keller, Karl Gruber, Klaus Zangger, Birgit Wiltschi

**Affiliations:** 1 Graz University of Technology, Institute of Molecular Biotechnology, Petersgasse 14, 8010 Graz, Austria; 2 Institute of Molecular Biosciences, University of Graz, Humboldtstraße 50, 8010 Graz, Austria; 3 Institute of Chemistry, University of Graz, Heinrichstrasse 28, 8010 Graz, Austria; 4 Austrian Centre of Industrial Biotechnology, Petersgasse 14, 8010 Graz, Austria

## Abstract

The enzyme 4-oxalocrotonate tautomerase shows remarkable catalytic versatility due to the secondary amine of its N-terminal proline moiety. In this work, we incorporated a range of proline analogues into the enzyme and examined the effects on structure and activity. While the structure of the enzyme remained unperturbed, its promiscuous Michael-type activity was severely affected. This finding demonstrates how atomic changes in a biocatalytic system can abolish its activity. Our work provides a toolbox for successful generation of enzyme variants with non-canonical catalytic proline analogues.

## Introduction

**Figure 1:**
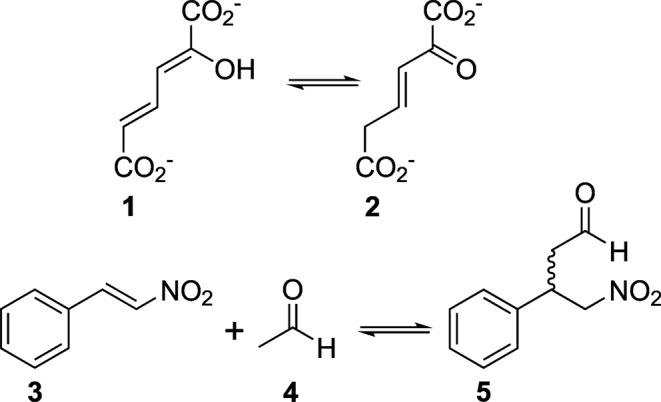
Natural (top) and promiscuous (bottom) reactions catalysed by 4-OT.

The enzyme 4-oxalocrotonate tautomerase (4-OT, EC 5.3.2.6) catalyses the tautomerisation of **1** to **2** in the breakdown of aromatics in *Pseudomonas putida*. An important promiscuous activity is its ability to catalyse carbon-carbon bond formation as shown in Fig. 1^1^.

**Figure 2:**
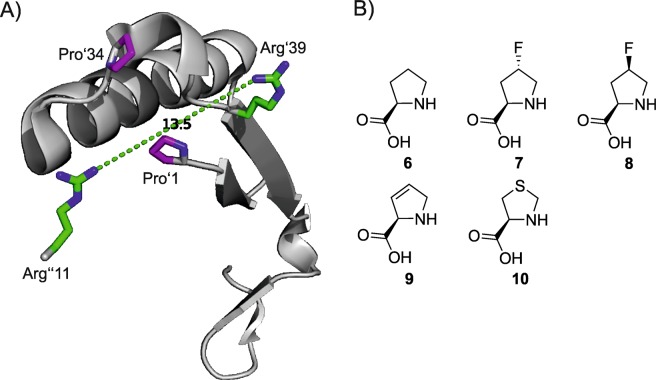
The structure of 4-OT and proline analogues used in this study. (**A**) Structural makeup of the active site of 4-OT (PDB 4X19). The arginine to the left (Arg”11) is contributed by the neighbouring monomer. Prolines are coloured purple; the amino-terminal proline (Pro’1) is visible in the centre. An arginine claw (green) is responsible for the correct orientation of the substrate with the two arginine residues being 13.5Å away from each other. (**B**) The following proline analogues were chosen for incorporation. **6**: (2*S*)-proline; **7**: (2*S*,4*R*)-4-fluoroproline; **8**: (2*S*,4*S*)-4-fluoroproline; **9**: (2*S*)-3,4-dehydroproline; **10**: (4*R*)-1,3-thiazolidine-4-carboxylic acid.

It stereoselectively catalyses the asymmetric Michael-type addition of various aldehydes to nitrostyrene **3** by employing its N-terminal proline in a manner reminiscent of amine organocatalysts. The resulting products are of interest as precursors in the synthesis of GABA analogues^2,3^. The hexameric enzyme exhibits a characteristic “arginine claw”^4^ in its active site (Fig. 2A) that is essential for the stabilisation of substrates in the natural and promiscuous activity. Similar to amine organocatalysts^5^, the secondary amine of the catalytic proline has been suspected to form an enamine-intermediate with aldehydes during the course of the reaction^6^. It is therefore absolutely essential for the promiscuous Michael-type activity of 4-OT. The supposed enamine formation is enabled by the hydrophobic environment in the active site, which lowers the pKa of proline’s secondary amine to 6.4^7^ as compared to 10.5^8^ of the free amino acid. The lowered pKa of the amine allows it to act as a general base as it exists largely in the uncharged state at physiological pH^7,9,10^. Mutation studies have shown that the secondary amine of proline is irreplaceable and that the enzyme’s activity is severely reduced if the N-terminal residue is any other than proline^11,12^. Due to this limitation to modify the active residue and inspired by the success of proline-like molecules in organocatalysis, we set out to incorporate a range of non-canonical proline analogues into 4-OT. We hoped to be able to modify and improve the industrially interesting Michael addition activity in an unprecedented manner by changing the active residue’s electronic and steric properties by atomic mutation. Since 4-OT only has a total of two proline residues (at positions 1 and 34, both depicted in Fig. 2A) in its short 62 amino acid sequence, we expected only minor disturbances in the structure upon proline analogue incorporation. Observed effects on the activity could thus be attributed mainly to the exchange of the N-terminal moiety. The analogues shown in Fig. 2B were chosen for incorporation because of their variety in electronic and steric properties. We took care to keep the differences subtle, such as a single atom exchange (analogues **7**, **8**) or bond saturation (residue **9**), to avoid gross perturbance of the protein structure.

There are two general ways to incorporate non-canonical amino acids into proteins: a site-specific way via stop codon suppression^13,14^ and a residue-specific way, where all residues of a given amino acid in a protein are exchanged for its non-canonical analogue^15,16^. Since 4-OT only has two prolines in its sequence and site-specific ways for proline analogue incorporation are not yet fully developed^17^, we chose the latter strategy of residue-specific incorporation. In this work, we show the successful incorporation of various proline analogues as active site residues into the 4-OT enzyme and elucidate the resulting changes in the promiscuous Michael-type activity.

## Results and Discussion

The residue-specific labelling of 4-OT with the proline analogues shown in Fig. 2B required the expression of the protein in a proline auxotrophic strain of *Escherichia coli*, which was grown in minimal medium until proline depletion^18^. Upon depletion, the analog was added to the culture and expression was induced (Supplementary Fig. S1). All variant proteins were produced in titers of 15 to 30 mg per litre of bacterial culture. Since the N- or C-terminal fusion with His- and Strep-purification tags completely abolished the enzyme activity, we used a modification of the purification procedure laid out by Zandvoort and coworkers^9^. This became necessary because the variant proteins showed a very dissimilar behaviour in the hydrophilic interaction chromatography with the published procedure, thus preventing the use of the same simple purification protocol for each variant. The modified purification scheme is made up of only three steps instead of the original five and allowed us to purify all variants with a single protocol (Supplementary Fig. S2). All variants were of a purity greater than 95% according to SDS-PAGE and densitometric analysis (Supplementary Fig. S3). We confirmed the incorporation of the proline analogues using HPLC-ESI-MS (Table 1). In our first attempt to incorporate proline analogues into 4-OT, we observed two key issues: Firstly, the incorporation was incomplete. We observed a mixture of protein species with either one or two proline analogues incorporated. Secondly, the amino-terminal methionine was not cleaved off completely (efficiency 75–80%) when the analogue at the ensuing position was structurally too different from the canonical proline. This was the case for analogues **7** and **8** (see Table 1).

**Table 1:** Mass determination of 4-OT variant preparations by HPLC ESI-MS.

Proline analogue	Number of substitutions / N-terminus	m_calc_	without MAP/ProS		with MAP/ProS	
			m_meas_	RA [%]	m_meas_	RA [%]
4-OT-**6**	/ methionine	6937.74				
	/ native	6806.70	6806.69	100	6806.69	100
4-OT-**7**	0 / native	6806.70	6806.68	21		
	1 / methionine	6955.74	6955.71	6		
	2 / methionine	6973.74	6973.70	18		
	1/native	6824.69	6824.68	27		
	2 / native	6842.68	6842.68	28	6842.68	100
4-OT-**8**	1 / methionine	6955.74				
	2 / methionine	6973.74	6973.66	20		
	1 / native	6824.69				
	2 / native	6842.68	6842.63	80	6842.68	100
4-OT-**9**	1 / methionine	6935.66				
	2 / methionine	6933.58				
	1 / native	6804.62				
	2 / native	6802.54	6802.63	100	6802.67	100
4-OT-**10**	0 / native	6806.70	6806.71	16		
	1 / methionine	6955.81				
	2 / methionine	6973.87				
	1 / native	6824.76	6824.67	31		
	2 / native	6842.83	6842.62	53	6842.61	100

The 4-OT variants were expressed with and without MAP/ProS helper plasmid and the obtained protein species were characterized. Samples were >95% pure as determined by SDS-PAGE (Supplementary Fig. S3) and densitometric analysis. Protein concentration (0.2–0.6 mg/mL) was determined by absorption measurement at 205 nm. m_calc_, calculated mass of the species; m_meas_, measured mass; RA, relative abundance.

The first phenomenon is caused by the low affinity of prolyl-tRNA synthetase (ProS) for proline analogues and can be alleviated by coexpression of ProS during proline analogue incorporation^19,20^. As for the second observation, we assumed that the base-level activity of the host methionine amino peptidase (MAP) was too low since most of the protein could be processed, but not completely. This could be solved by overexpressing MAP. We constructed a helper plasmid to express both ProS and MAP in a single operon under the weak constitutive EM7 promoter (for plasmid map see Supplementary Fig. S4A). After confirming the expression of MAP and ProS under expression conditions used for Pro analogue incorporation (Supplementary Fig. S4B), we co-transformed the proline auxotrophic strain with the 4-OT expression plasmid and the helper plasmid and repeated the expression of 4-OT under proline analogue incorporation conditions. The protein variants obtained with the helper plasmid present exhibited full incorporation of proline analogues and complete processing of the N-terminal methionine (Table 1).

**Figure 3:**
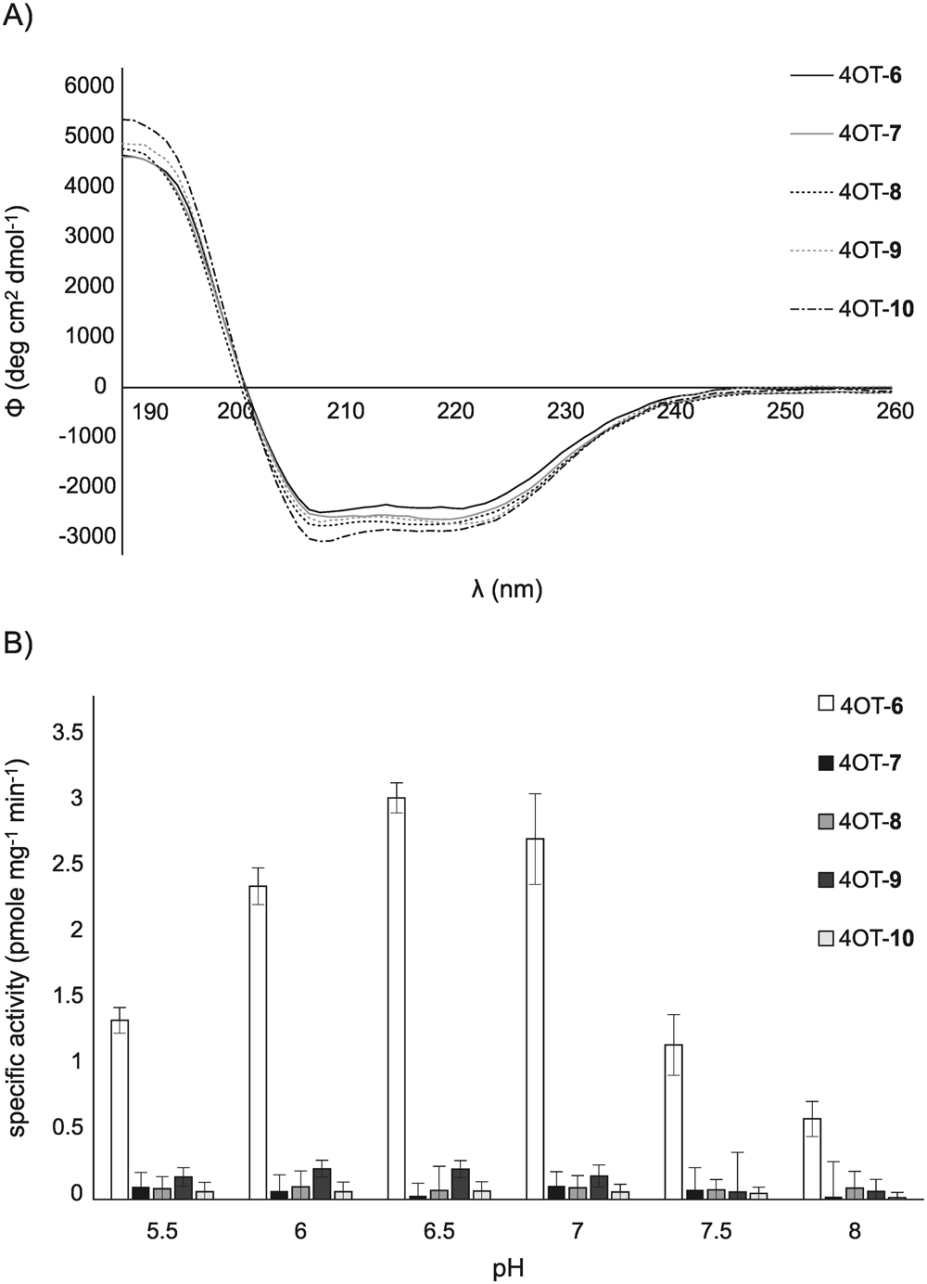
Secondary structure and catalytic activity of the 4-OT variants. (**A**) Circular dichroism spectra of 4-OT and the variant proteins (0.2 mg/mL each) in 50 mM phosphate buffer, pH 8 at room temperature. (**B**) Activity measurements of 4-OT and the variant proteins. Depletion of nitrostyrene **3** in the presence of acetaldehyde in 50 mM phosphate buffer was determined spectrophotometrically at 320 nm. 4-OT loses its hexameric structure below pH 5.5^7^. The mean of at least two technical replicates is shown, error bars represent the standard deviation. Samples used for both experiments were >95% pure as determined by SDS-PAGE and densitometric analysis (see Supplementary Fig. S3).

To validate the integrity of the secondary and quaternary structures of the 4-OT variants, we recorded circular dichroism spectra (Fig. 3A). The spectra of all variants were essentially identical, and they also correlated with previously published spectra recorded by Cisneros and coworkers^21^. Our finding, particularly in the context of NMR and structural data (*vide infra*), hints at the structural integrity of all variants, which suggests that full incorporation of the analogues did not perturb the structure of 4-OT.

A different picture arose when we looked at the activity of 4-OT and its variants. Only the wildtype protein containing proline showed significant activity in the Michael-type addition reaction, whereas the variant proteins containing the different proline analogues showed surprisingly severely reduced activity under all tested pH conditions (Fig. 3B). Even the structurally very similar dehydroproline **9**, which differs from proline only in the presence of a double bond in the ring structure, could not preserve the enzyme activity.

One possible explanation for the loss in Michael-type activity is the change in protonation state of the secondary amine in the different analogues. Theoretical prediction^22^ of secondary amine pKa’s for analogues give 9.7 for **7** and **8**, 10.7 for **9**, and 7.7 for **10** in comparison to proline at 10.6. The pKa of proline is lowered by approximately 4 units^7^ in the active site of 4-OT compared to the free amino acid. If the analogues’ pKa’s were comparably lowered in the corresponding 4-OT variants, **7**, **8** and **9** would behave very similarly to proline (estimated pKa’s ~5.7–6.7 *vs* pKa_Pro_ 6.4)^7^ and only the pKa of **10** would be substantially lower (estimated pKa ~3.7 *vs* pKa_Pro_ 6.4)^7^. Even though the analogues exhibit different pKa’s, changes in pKa of the secondary amine are not very likely responsible for the loss in activity because no variant exhibited significant changes in activity at different pH values, except for dehydroproline **9** and here the differences were very minor (Fig. 3B).

This led us to the question whether the substitution of the catalytic proline at position 1 or of the structural proline at position 34 was responsible for the loss in activity. Conservation of proline 34 in the homologues of 4-OT (Supplementary Fig. S5) suggests an important role for this residue in the structural and/or functional integrity of 4-OT. Mutagenesis with concomitant activity screening should give a clear picture of its role in activity. We therefore constructed an exhaustive mutation library of the 4-OT gene at position 34 and used a whole cell screening assay to identify active variants^23^. Screening the library gave a hit for a mutant 4-OT P34E with glutamate at position 34 that maintained similar Michael-type activity and expression levels as wildtype 4-OT. This result was recently confirmed in a study by van der Meer and coworkers^24^. Upon incorporation of proline analogues, however, the variants of 4-OT P34E also lost all of their Michael-type activity, similar as the variants of wildtype 4-OT. This strongly indicated that the catalytic proline at position 1 was the culprit and that it could not be replaced by any of the analogues.

**Figure 4:**
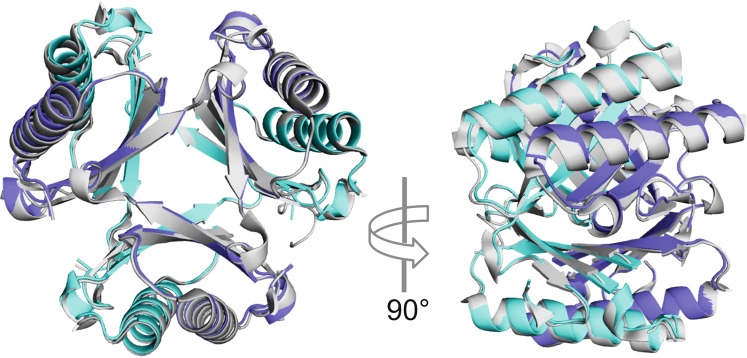
Superposition of a 4-OT-**9** (PDB 6GHW) and a 4-OT (PDB 4X19)^6^ hexamer. 4-OT is shown in grey.

These results warranted further investigation into the structural and dynamic make-up of 4-OT with substituted prolines. We chose again the 4-OT variant with analogue **9** incorporated (4-OT-**9**) for further investigation and solved its crystal structure. The crystal structure (Fig. 4; Supplementary Fig. S6; crystallographic data are listed in Supplementary Table S1) suggests that there is no significant difference between the tertiary structures of 4-OT and 4-OT-**9**. The structures are virtually identical and even the residues in the active site show no deviation from the wild-type reference structure.

**Figure 5:**
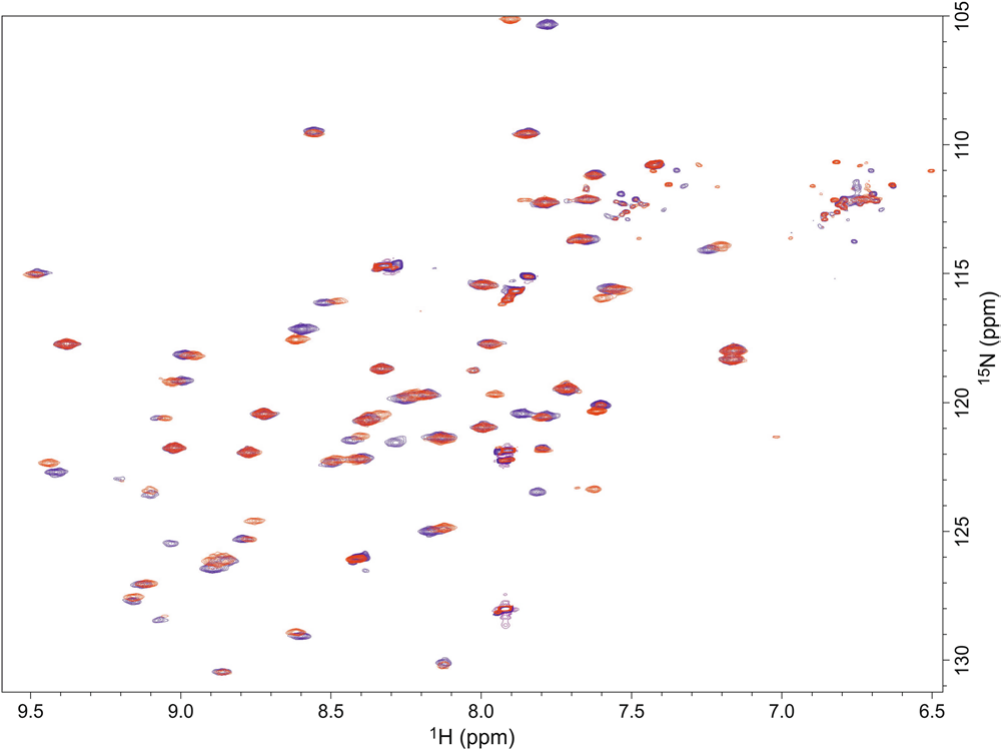
Overlay of ^1^H-^15^N HSQC spectra of 4-OT (blue) and 4-OT-**9** (red).

However, crystal structures only give a static, frozen image of a protein in a rather unnatural state so that it is very possible that differences of the protein structures in solution and their dynamics make the difference in catalytic activity. We therefore performed ^1^H-^15^N-HSQC NMR spectroscopy to detect changes in protein solution structure. The ^1^H-^15^N-HSQC spectra of 4-OT^25^ and 4-OT-**9** look very similar (Fig. 5). Both indicate a well-structured single conformation protein. Small differences of some peaks are expected for the single amino acids exchange. Based on the high similarity of peak numbers, positions and line-width, no huge changes in structure or protein flexibility can be expected. To get some more quantitative information on protein mobility, we determined the overall {^1^H}^15^N-NOEs and deviations between wild-type and 4-OT-**9**. For 4-OT and 4-OT-**9**, the average {^1^H}^15^N-NOE for all residues were 0.73 ± 0.23 and 0.72 ± 0.24, respectively. Considering the higher flexibility near the termini, these averaged overall values are typical for well-structured proteins. The average difference of hetero NOEs of individual NMR signals, between 4-OT and 4-OT-**9** was only 0.05. To look for differences in the region affected by the amino acid exchanges, the hetero NOEs of only the signals which show chemical shift differences upon mutation were also compared. The average difference of hetero NOEs of those residues which show chemical shift changes between 4-OT and 4-OT-**9**, so for residues close to residues 1 and 34, is only 0.03. This indicates that even around the site of incorporation, there is no measurable change in flexibility.

These results strongly support the hypothesis that substitution of the N-terminal catalytic proline for any of the analogues was responsible for the adverse effects on activity. Since the analogues are structurally very similar to proline we suspect that the loss in Michael-type activity is mainly due to changes in the fast dynamics of the 4-OT active site. In the crystal structure of 4-OT in complex with *trans*-β-nitrostyrene **3**, the secondary amine of proline is perfectly oriented towards the double bond of **3** where the addition of acetaldehyde takes place^24^. It is very likely that this precise orientation is crucial for the Michael-addition activity of 4-OT and it might be disturbed by the substitution of proline with its analogues.

Our work shows once again that nature is very efficient at evolving catalysts, where minute structural alterations can lead to significant effects on the catalyst’s functionality. 4-OT evolved to perfectly use the unique catalytic capabilities of proline. Even for its promiscuous Michael-type activity, it does not allow any structural or chemical deviations of this residue. Nevertheless, we showed for the first time the successful and complete incorporation of proline analogues into a representative of the tautomerase superfamily. This opens up a new exciting research field, where members of the family and other proteins containing catalytic proline residues can be screened for and tailored to new functionalities using proline analogues.

## Methods

### Plasmid construction

Enzymes were from Thermo Fisher Scientific (Waltham, MA) unless indicated otherwise. To construct plasmid pQE80L-4OT, the backbone plasmid pQE80L (Qiagen, Hilden, Germany) was doubly digested with SphI/PstI and gel purified with the Promega (Madison,WI) Wizard® SV Gel and PCR Clean-Up System. The insert was prepared by colony PCR of *Pseudomonas putida* mt-2 (KT2240 from the German Collection of Microorganisms and Cell Cultures, DSMZ) using the primers 4OT_Fwd and 4OT_Rev (Supplementary Table S2). The PCR product was purified using the Wizard® SV Gel and PCR Clean-Up System, and was inserted into restricted pQE80L using Gibson^26^ isothermal assembly. The MAP/ProS helper plasmid (see the plasmid map in Supplementary Fig. S4A) was constructed as follows: The genes encoding MAP and ProS were amplified using MAP_Fwd/MAP_Rev and ProS_Fwd/ProS_Rev as primers in a PCR reaction with *E. coli* MG1655 (Coli Genetic Stock Center CGSC, Yale University, New Haven, CT) genomic DNA as the template. The genomic DNA was prepared by the method of Lõoke *et al*.^27^. The resulting PCR products were gel purified as described above and subcloned separately for amplification. The gene encoding ProS was amplified from the vector using ProS_G_Fwd and ProS_G_Rev. The product was Gibson-cloned into the previously restricted vector containing MAP. To obtain the final helper plasmid, three PCR fragments were generated to be assembled into a single vector using Gibson cloning. Primers F1 Fwd/F1 Rev were used to amplify the kanamycin resistance marker and the p15A origin of replication from NEB pACYC177 plasmid. Primers F2 Fwd/F2 Rev amplified the EM7 promoter from the pEM7/Zeo plasmid (Invitrogen, Carlsbad, CA) and F3 Fwd/F3 Rev were used to amplify the MAP/ProS construct. The primers are listed in the Supplementary Table S2.

### Expression of 4-OT with and without the MAP/ProS helper plasmid

The same expression protocol was used for strains with and without the MAP/ProS helper plasmid. To create the expression strain, the proline auxotrophic *E. coli* strain BWEC44 (genotype: B F^−^ *ompT hsdS*_B_(r_B_^−^ m_B_^−^) *dcm*^+^ *gal* λ(DE3) Tet^r^ *endA proC*Δ0; the generation of this strain will be described elsewhere) was transformed with pQE80L-4OT alone or it was co-transformed with pQE80L-4OT and the MAP/ProS helper plasmid. Single colonies were inoculated into 10 mL Luria-Bertani (LB) medium with either 100 mg/L ampicillin or 100 mg/L ampicillin and 50 mg/L kanamycin and incubated over night with shaking at 37 °C. The next day, the overnight cultures were spun down at 4000 g for 10 min and resuspended in sterile 0.9% NaCl solution. M9 expression medium was prepared by mixing 200 mL sterile 5x M9 stock (33.9 g/L Na_2_HPO_4_, 15 g/L KH_2_PO_4_, 5 g/L NH_4_Cl, 2.5 g/L NaCl), 20 mL glucose (1 M), 1 mL 1 M MgSO_4_, 1 mL 1 M CaCl_2_, 60 µL trace elements (consisting of 40 g/L FeSO_4_ × 7 H_2_O, 10 g/L MnSO_4_, 10 g/L AlCl_3_ × 6 H_2_O, 7.3 g/L CoCl_2_ × 6 H_2_O, 2 g/L ZnSO_4_ × 7 H_2_O, 2 g/L Na_2_MoO_4_ × 2 H_2_O, 1 g/L CuCl_2_ × 2 H_2_O, 0.5 g/L H_3_BO_4_, 414 mL/L HCl conc.) and 40 mL 60 g/L yeast extract in a 2 L baffled shake flask. This was brought to 1 L total volume by adding sterile doubly-distilled water (ddH_2_O). The medium was then inoculated with the overnight culture at a D_600_ of 0.2. The baffled flask was placed in a shaking incubator at 37 °C and 180 rpm and the D_600_ of the culture was monitored continuously. Proline depletion was reached when the D_600_ of the culture did not increase for 30 min. At this point, 1 mM proline or proline analogue and 0.5 mM IPTG were added and the culture was transferred to 28 °C with shaking at 180 rpm. The protein expression continued for 5 h before the culture was harvested by centrifugation (Beckmann-Coulter JA-10 rotor, 5000 rpm, 30 min, 4 °C).

### Purification of 4-OT

The cell pellet was washed in 50 mL Buffer A (10 mM NaH_2_PO_4_, pH 8) and again pelleted (4300 g, 30 min, 4 °C). The cell pellet was resuspended in 15 mL Buffer A and the suspension was sonicated on ice (5 × 2 min pulses) using the Branson (Danbury, CT) Sonifier®. The crude lysate was cleared by centrifugation (Beckman-Coulter JA25.50 rotor, 20000 rpm, 30 min, 4 °C). The lysate was made 1.6 M in (NH_4_)_2_SO_4_ by adding the solid salt and letting it dissolve slowly overnight at 4 °C on a spinning wheel. The resulting suspension was again cleared of protein debris by centrifugation (Beckman-Coulter JA25.50 rotor, 20000 rpm, 30 min, 4 °C). To remove (NH_4_)_2_SO_4_, the cleared lysate was subjected to gel filtration on a HiPrep 26/10 column (GE Healthcare, Vienna, Austria) using an Äkta pure FPLC system (GE Healthcare). The column was conditioned with Buffer A. The lysate was then loaded with at a flow of 10 mL/min. Buffer A was then used without changing the flow rate to elute the proteins from the column. Protein elution was monitored via UV absorption at 280 nm and the protein-containing eluate was collected. The collected eluate was then loaded onto a 25 mL DEAE FF ion exchange column (GE Healthcare; self-packed in a XK 26/40 column also from GE Healthcare) previously equilibrated with Buffer A. After loading, the column was left for 15 min to allow the proteins to bind properly. The column was then washed with Buffer A until no protein elution was detectable at 280 nm. Gradient elution was then carried out for 7 column volumes from Buffer A to Buffer B (10 mM NaH_2_PO_4_, 90 mM Na_2_SO_4_, pH 8) with a flow rate of 2 mL/min. 2 mL fractions were collected in 96 deep well plates using a fraction collector. The UV absorption at 205 nm was recorded to identify the fractions containing 4-OT. The protein content of those fractions was determined spectrophotometrically by UV-absorption at 205 nm.

### SDS-PAGE and protein purity determination

SDS-PAGE gels were 12% (GenScript, Piscataway, NJ). Samples were prepared by adding 4x loading buffer (200 mM Tris-HCl pH 6.8, 40% glycerol, 8% SDS, 400 mM DTT, 0.4% bromphenol blue) and heating to 70 °C for 15 min. Gels were run for 55 min at 140V in 1x MES buffer (6.06 g/L Tris base, 9.76 g/L MES, 1.0 g/L SDS, 0.3 g/L EDTA) and stained with Coomassie Blue (0.1% (w/v) in 50% (v/v) methanol, 10% (v/v) glacial acetic acid, 40% (v/v) H_2_O). For purity determination, gels were scanned and loaded into the GelAnalyzer2010a software. The lanes were defined, and the background was subtracted via the baseline method. Bands were detected automatically, and the percentage was calculated via the peak volumes.

### Activity measurements of 4-OT

The activity measurements were carried out in 200 µl volume in a Greiner UV-Star 96-well microplate. The reaction consisted of 15 µl *trans*-β-nitrostyrene (10 mM in abs. EtOH), 7.5 µl acetaldehyde (1 M in ddH_2_O), 87.5 µl reaction buffer (50 mM NaH_2_PO_4_, varying pH) and 1 mg/mL enzyme solution (in Buffer A). The reaction was prepared at room temperature and acetaldehyde was added to start the reaction just before starting the measurement. The reaction was monitored on a BioTek Eon plate reader (Winooski,VT) by following the depletion of *trans*-β-nitrostyrene at 320 nm.

### Circular dichroism spectroscopy

4-OT and its variants were rebuffered into reaction buffer (50 mM NaH_2_PO_4_, pH 7.5) using PD-10 columns (GE Healthcare, Chicago, IL) and all samples were brought to 0.2 mg/mL concentration as determined by absorption measurement at 205 nm. Far-UV CD spectra (190–260 nm) were then recorded at room temperature on a J-715 CD spectropolarimeter employing a 1 mm quartz cuvette (JASCO, Tokyo, Japan).

### Generation and screening of a 4-OT Pro34 mutant library

pQE80L-4OT was digested with EcoRI and HindIII to remove the 4-OT insert and then gel purified as described above. A PCR reaction was set up with primers 4OT34ProNNK_Fwd and 4OT34ProNNK_Rev to generate an NNK library of the codon at position 34. Primers 4OTGibson_Fwd and 4OTGibson_Rev (Supplementary Table S2) were used as flanking primers in the PCR reaction. The resulting product was gel purified as described above. A Gibson assembly reaction was set up with the digested vector and the PCR product. The reaction was electroporated into proline auxotrophic *E. coli* BWEC44 and the resulting clones were used for activity screening.

Deep-well microplates were filled with 200 µL M9 medium supplemented with 4 g/L yeast extract, 1 mM proline and 100 µg/mL ampicillin. A total of 3 plates was inoculated with *E. coli* BWEC44 blank strains carrying an empty pQE80L plasmid and library clones. The plates were incubated at 37 °C and shaking at 250 rpm overnight. The next day, 3 deep-well plates were filled with 750 µL of the identical medium and inoculated with 25 µL overnight culture of the corresponding wells. The plates were again incubated at 37 °C for 3.5 h and then moved to 28 °C with shaking at 320 rpm before induction with 0.5 mM IPTG. The cells were incubated for expression at 28 °C and 350 rpm overnight. Plate cultures were harvested by centrifuging for 15 min at 4000 × g. Whole cells were then resuspended in 250 µL reaction buffer (10 mM NaH_2_PO_4_, pH 6.4, 1 mM *trans*-β-nitrostyrene in 10% (v/v) EtOH). Acetaldehyde was added, and the cells were left to react at room temperature at 800 rpm shaking for 30 min. Whole-cell reaction mixtures where then spun down again at 4000 g for 15 min. The supernatants were collected and transferred to UV-microplates. To be able to correct for accidentally resuspended and transferred cells, plates were also read at 600 nm to measure the cell density. The clones which gave the most appropriate 320/600 nm absorption ratio were sequenced.

### Crystal structure determination

Screening for crystallization conditions was performed with an Oryx8 robot (Douglas Instruments, Hungerford, UK) using Morpheus Screen MD 1–46 (Molecular Dimensions, Los Angeles, CA) by the sitting drop vapor-diffusion method in 96-well Swissci plates (Molecular Dimensions). Drops contained 0.5 μl protein (6 mg/mL in 0.1 M PCTP buffer pH 7.0), incubated for 30 min with 3 mM *trans*-β-nitrostyrene dissolved in DMSO) and 0.3 μl of screening solution. The crystallization plates were incubated at 289 K. Initial crystals were obtained within 1–4 weeks in several crystallization conditions. Crystal optimizations were performed by serial dilution of Morpheus conditions A9, B9, D9, E9, F9, G9 and different drop ratios. Before freezing in liquid nitrogen, additional *trans*-β-nitrostyrene dissolved in DMSO was added to some of the drops. Data collection was performed at the ESRF (ID30B and ID23-2 beamlines, Grenoble, France) at 100 K. Crystals diffracting to 2.3 Å were obtained in 88% of 1–45 Morpheus condition (0.12 M alcohols, 0.1 M Tris (base), BICINE pH 8.5, 50% (v/v) precipitant mix composed of 40% (v/v) PEG 500 MME; 20% (w/v) PEG 20000). Data were processed and scaled using the XDS program package^28^. The structure was solved by molecular replacement using Phaser^29^. Manual rebuilding was performed in Coot^30^ and refinement using the PHENIX software suite^31^. The structure of the native 4-OT from *Pseudomonas putida* mt-2 (PDB: 4X19)^6^ was used as a search template. A randomly chosen set of 5% of the reflections was used for R_free_ calculations^32^. There are 4 chains present in the asymmetric unit. The electron density is clearly defined only for 3 chains. Density for the forth chain is visible but unordered. Therefore, this chain could not be built and is not included in the final structure. The additional density for *trans*-β-nitrostyrene was not observed. The stereochemistry and geometry were analysed using MolProbity^28^. In the Ramachandran plot, all residues of the model are located in the most favourable or allowed regions. The summary of the data collection and processing statistics are available in Supplementary Table S1. The atomic coordinates and structure factors have been deposited in the Protein Data Bank as entry 6GHW. All structure-related pictures were generated with PyMOL^33^.

### NMR

^15^N-labelled 4-OT and 4-OT-**9** were expressed and purified as described above except for the substitution of NH_4_Cl by ^15^NH_4_Cl in the growth medium. For NMR acquisitions, ^15^N-labelled 4-OT or 4-OT-**9** were dialyzed into 50 mM potassium phosphate buffer pH 6.5 (containing 0.02% NaN_3_) to final concentrations of 0.3 mM (4-OT) and 0.3 mM (4-OT-**9**). To each sample 10% ^2^H_2_O was added for field-frequency locking. All spectra were recorded at 300 K on a Bruker Avance III 700 MHz NMR spectrometer (Bruker, Billerica, MA), equipped with a 5 mm cryogenically cooled TCI probe with z-axis gradients. Two-dimensional ^1^H-^15^N-HSQCs were acquired with data matrices of 2048 × 256 data points. Thirty-two scans were recorded for each increment and 60° phase shifted squared sine-bell window functions were applied in both dimensions prior to Fourier transformation using TopSpin 3.1. The {^1^H}–^15^N heteronuclear NOEs were extracted from ^1^H-saturated and unsaturated spectra (3 s saturation time) ^1^H-^15^N HSQC-type spectra after processing using NMR-Pipe^34^ and integration with NMRView^35^.

## Supplementary information

Supplementary Information

## Data Availability

All data generated or analysed during this study are included in this published article (and its Supplementary Information File).
