# Supplementary material for: Substituting the catalytic proline of 4-oxalocrotonate tautomerase with non-canonical analogues reveals a finely tuned catalytic system
Source: Sci Rep. 2019 Feb 25;9:2697. doi: 10.1038/s41598-019-39484-9 (PMC6389900; doi:10.1038/s41598-019-39484-9)
Supplement: Supplementary file 1 — Supplementary Information [file 41598_2019_39484_MOESM1_ESM.pdf]

Supplementary Information

Substituting the catalytic proline of 4-oxalocrotonate tautomerase with non-canonical analogues reveals a finely tuned catalytic system

Michael S. Lukesch[1], Tea Pavkov-Keller[2], Karl Gruber[2,4], Klaus Zangger[3] & Birgit Wiltschi[4]*

[1] Graz University of Technology, Institute of Molecular Biotechnology, Petersgasse 14, 8010 Graz, Austria

[2] Institute of Molecular Biosciences, University of Graz, Humboldtstraße 50, 8010, Graz, Austria

[3] Institute of Chemistry, University of Graz, Heinrichstrasse 28, 8010, Graz, Austria

[4] Austrian Centre of Industrial Biotechnology, Petersgasse 14, 8010 Graz, Austria

* Corresponding author; email: birgit.wiltschi@acib.at

Supplementary Figures and Tables

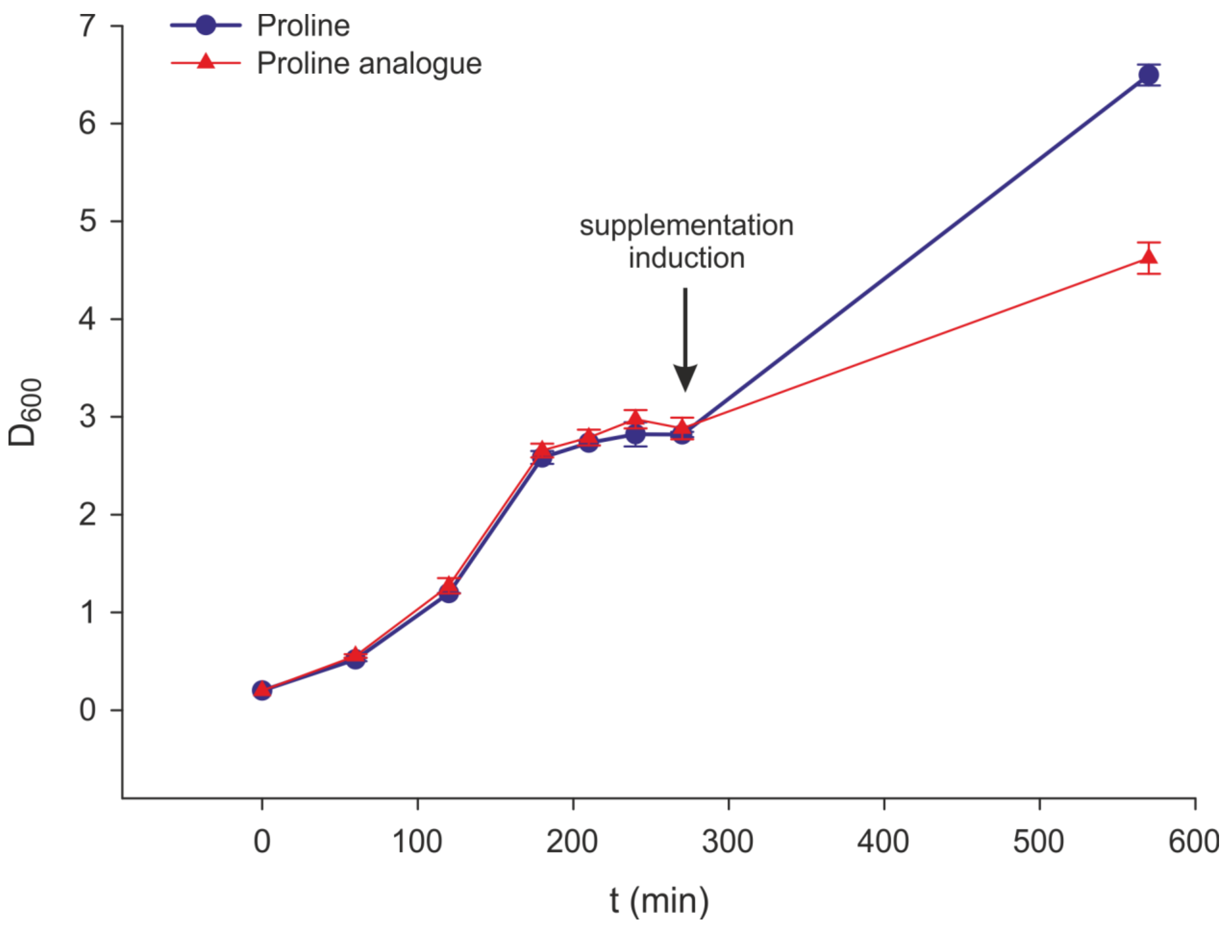

Figure S1. Growth of the proline auxotrophic *Escherichia coli* strain expressing 4-OT in a typical incorporation experiment. The strain was grown in M9 minimal medium containing a limited amount of proline until the depletion of the available proline. The analogue was then added, and the expression of 4-OT was induced. The apparent increase in D_{600} in the culture containing the analogue is most likely caused by the accumulation of inclusion bodies during overexpression since some of the 4-OT ended up in inclusion bodies according to SDS-PAGE.

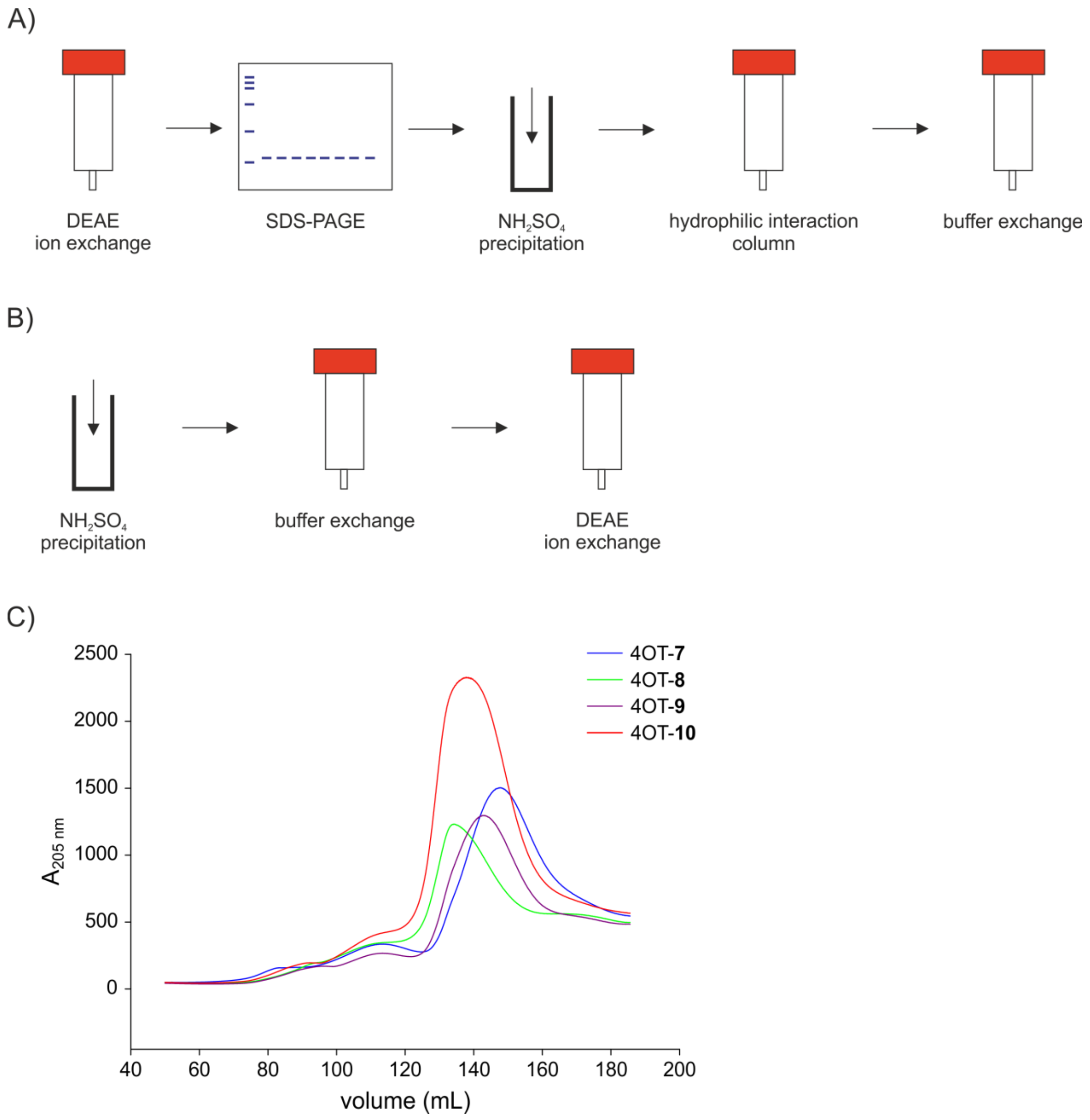

Figure S2. Purification of 4-OT. (**A**) Purification scheme for wildtype 4-OT as described by Zandvoort and coworkers[1] (**B**) The simplified purification scheme we devised for wildtype 4-OT and its variants. The initial lysate is precipitated with ammonium sulfate, followed by a buffer exchange. The desalted sample containing 4-OT is then subjected to ion exchange chromatography (IEX) and eluted in high purity. (**C**) Elution behaviour of the different variants on the final IEX column. Numbers refer to the analogues (see Fig. 2B in the main text) incorporated into the variants.

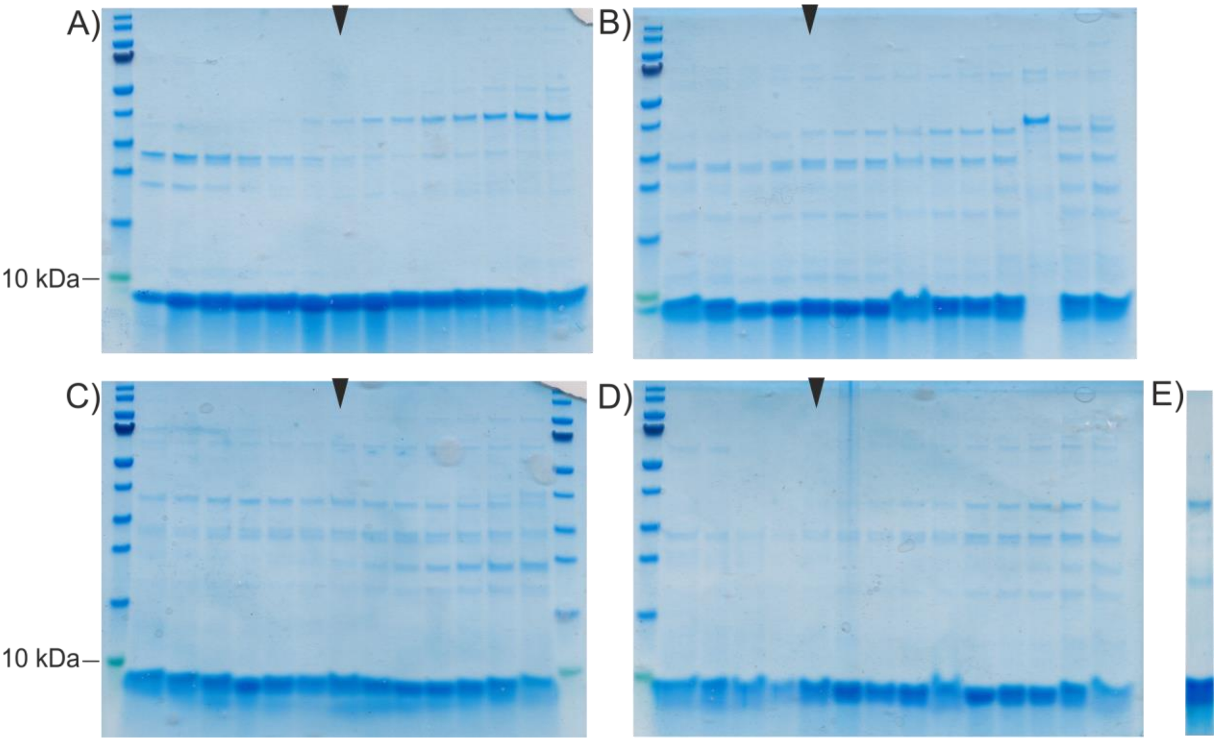

Figure S3. SDS-PAGE for densitometric analysis. (**A**) Fractions after IEX purification of wildtype 4-OT. Fractions after IEX purification of (**B**) 4-OT-**7**; (**C**) 4-OT-**8**; (**D**) 4-OT-**9**; and (**E**) single fraction of purified 4OT-**10**. The purity of the fractions used for protein analysis was determined using the GelAnalyzer software. Fractions used for analysis are marked by a black arrow. The numbers refer to the analogues incorporated into the variants (see Fig. 2B in the main text).

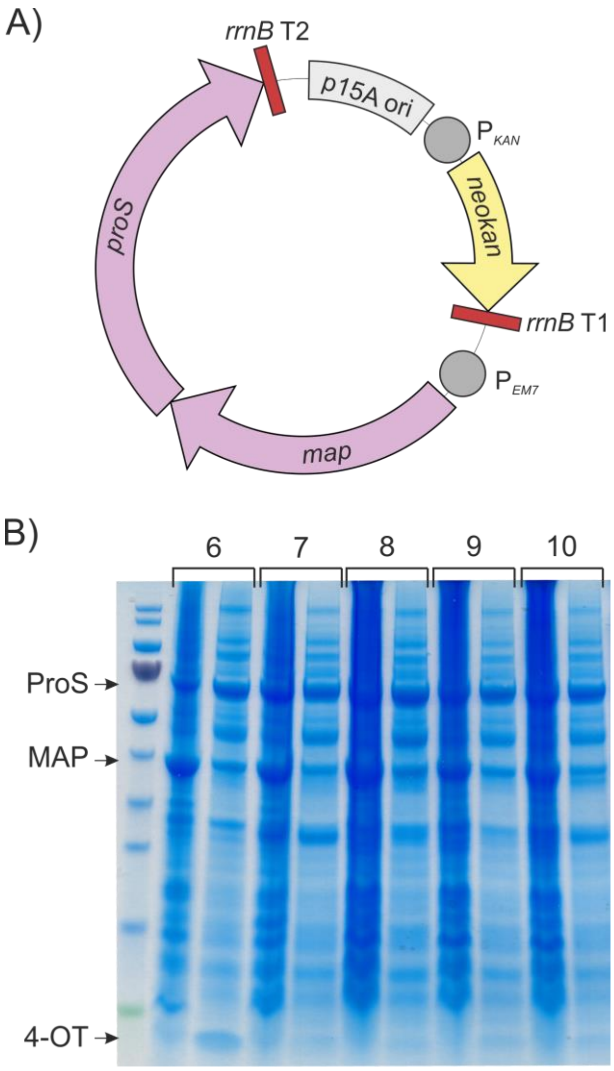

Figure S4. Expression of prolyl-tRNA synthetase ProS and methionine amino peptidase MAP. (**A**) Plasmid map of helper plasmid p15a MAP/ProS. (**B**) Expression of ProS/MAP and 4-OT with different analogues present. The left lane shows the insoluble protein fraction after sonication, the right shows the soluble fraction each. The numbers refer to the analogues incorporated into the variants (see Fig. 2B in the main text).

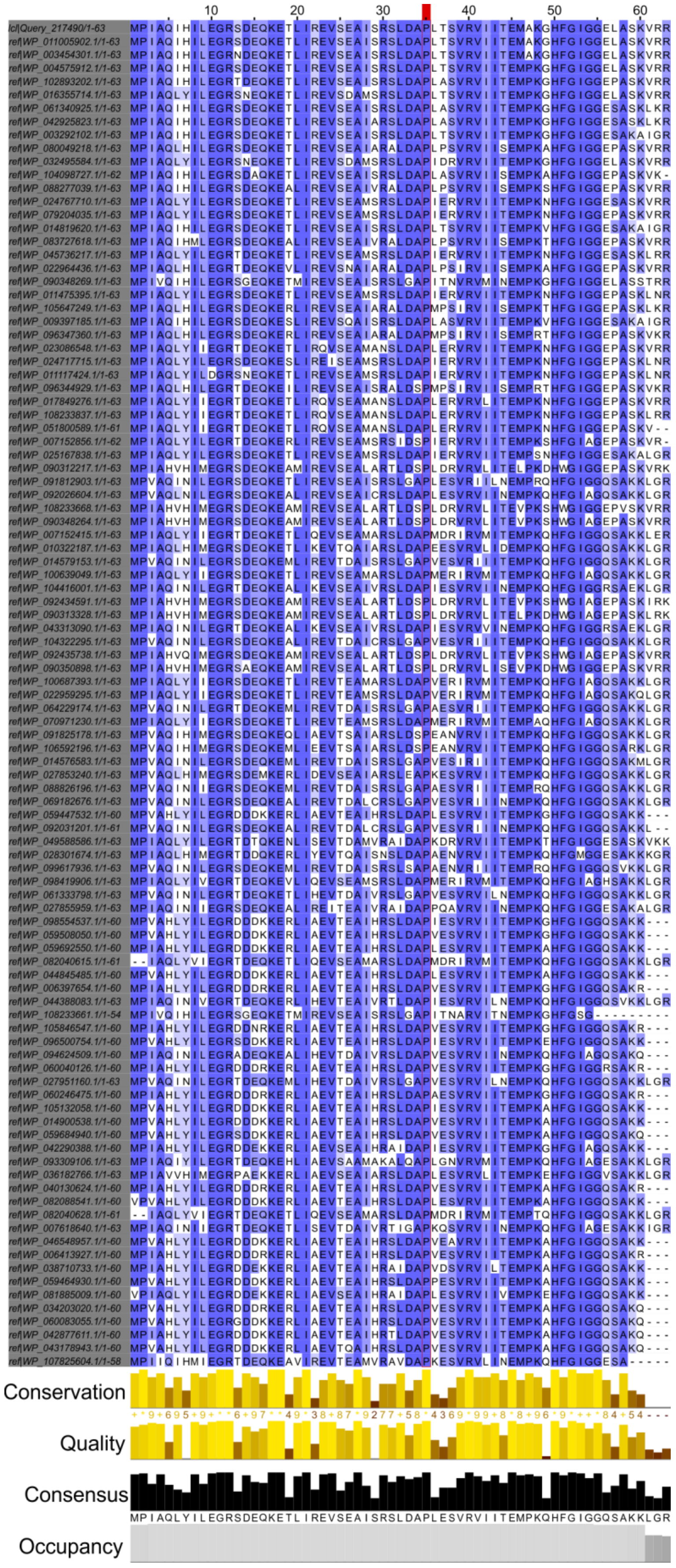

Figure S5. 4-OT (WP_011005902.1) homologues in the bacterial kingdom. Proline at position 34 (marked by the red square) is conserved in all homologues. The list was compiled using NCBI-BLAST and the image was generated with JalView[2].

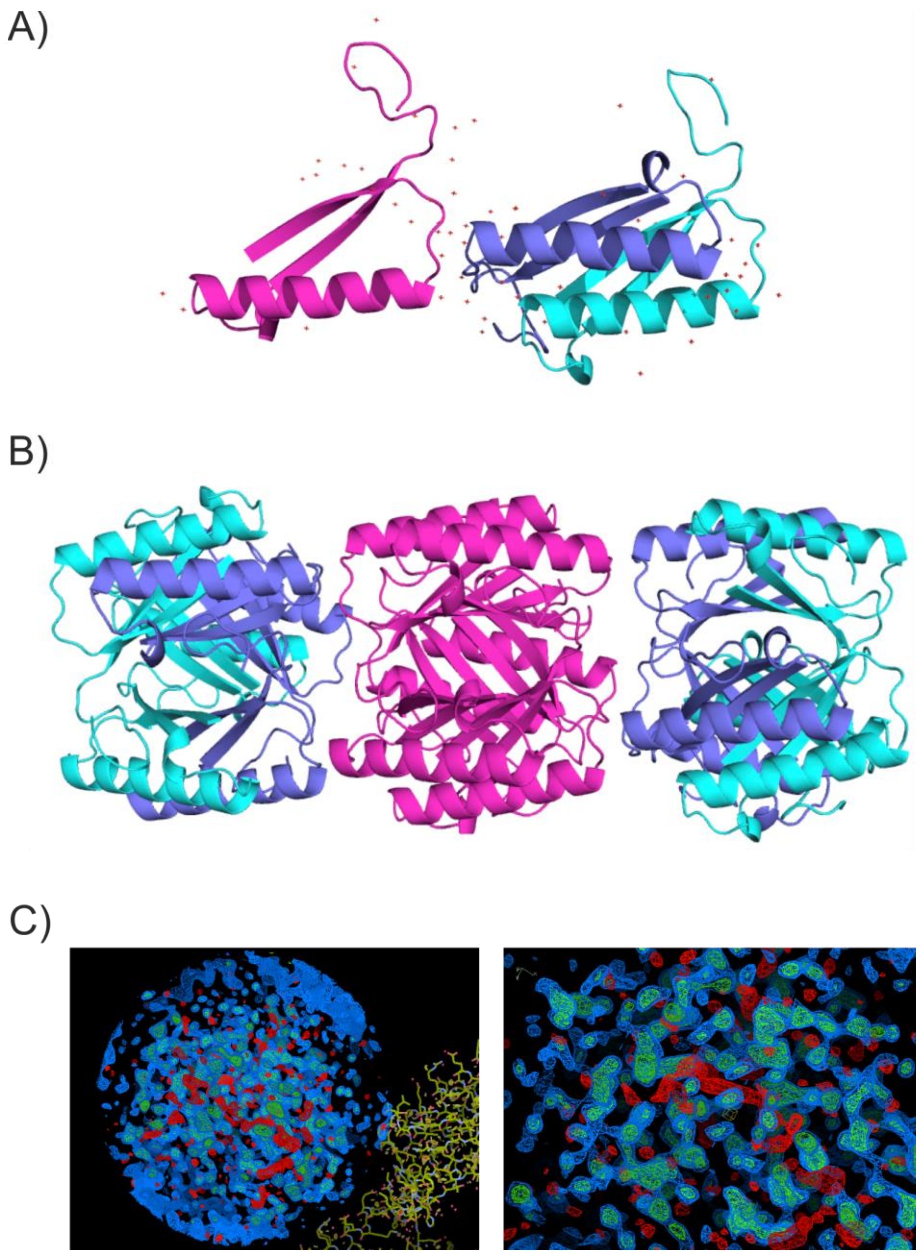

Figure S6 Structure analysis of 4-OT-**9**. (**A**) Three chains (magenta, cyan and blue) of 4-OT-**9** as present in the asymmetric unit. Chains are shown in cartoon representation and water molecules are shown in red spheres. (**B**) The hexamer of 4-OT-**9** obtained by generating symmetry molecules as present in the crystal structure. (**C**) Non-assigned electron density for the region where the fourth chain could be placed in the asymmetric unit. Generating the symmetry-related molecules, an additional hexamer could be accommodated.

Supplementary Tables

Table S1. Data collection and refinement statistics for 4-OT-**9** (PDB entry 6GHW). Statistics for the highest-resolution shell are shown in parentheses.

	**4-OT-9**
**Wavelength**	0.8731
**Resolution range**	34.4 - 2.3 (2.38 - 2.3)
**Space group**	*R* 3 2
**Unit cell (Å, °)**	85.30, 85.30, 155.46<br>90, 90, 120
**Total reflections**	26815 (1899)
**Unique reflections**	9550 (764)
**Multiplicity**	2.8 (2.5)
**Completeness (%)**	95.99 (78.26)
**Mean I/sigma(I)**	4.55 (1.53)
**Wilson B-factor**	27.37
**R-merge**	0.162 (0.541)
**R-meas**	0.199 (0.680)
**R-pim**	0.114 (0.405)
**CC1/2**	0.97 (0.62)
**CC***	0.99 (0.87)
**Reflections used in refinement**	9548 (763)
**Reflections used for R-free**	494 (43)
**R-work**	0.289 (0.336)
**R-free**	0.319 (0.367)
**CC(work)**	0.886 (0.620)
**CC(free)**	0.837 (0.717)
**Number of non-hydrogen atoms**	1368
**macromolecules**	1299
**ligands**	1
**solvent**	68
**Protein residues**	171
**RMS(bonds)**	0.003
**RMS(angles)**	0.46
**Ramachandran favored (%)**	100.00
**Ramachandran allowed (%)**	0.00
**Ramachandran outliers (%)**	0.00
**Rotamer outliers (%)**	1.48
**Clashscore**	2.67
**Average B-factor**	30.28
**macromolecules**	30.13
**ligands**	45.92
**solvent**	32.84

Table S2. Primers used in this study.

Primer	Sequence 5'→3'
4OT_Fwd	CAATTTCACACAGAATTCATTAAAGAGGAGAAATTAAGCATGCCTATTGCCCAGATCCAC
4OT_Rev	CTATCAACAGGAGTCCAAGCTCAGCTAATTAAGCTTGGCTGCAGTTATCAGCGTCTGACCTTGCTGGCC
MAP_Fwd	AATTGGTACCGGAAA AAGGAGATCTGCATATGGCTATCTCAATCAAGACCC
MAP_Rev	CCATTAATTAATTATTCGTCGTGCGAGAT TATC GCC
ProS_Fwd	AATTG CATGCGTACTAGCCAATACCTGCTCTCCA
ProS_Rev	TTAAAAGCTTTTATTAGCCTTTAATCTGTTTCACCA GATATTCGACG
ProS_G_Fwd	GATAATCTCGCACGACGAATAATTAATGAATTCATTAAAGAGGAGAAATTAAGCATGCGT
ProS_G_Rev	TTCTGCGTTCTGATTTAATCTTAATTTATTAGCCTTTAATCTGTTTCACCAGATATTCGACG
F1 Fwd	GCGCTAGCGGAGTGTATACTGG
F1 Rev	ATTCTAGAGACCCGTCGACCATCAGGCATCAAATAAAACG
F2 Fwd	CTGATGGTCGACGGGTCTCTAGAATTTAAATGCGACAATTAATCA
F2 Rev	TTGAGATAGCCATTAGTATATCTCCTTGAATTCGTTTAGTTCC
F3 Fwd	GGAGATATACTAATGGCTATCTCAATCAAGACCCC
F3 Rev	GTAAGCCAGTATACACTCCGCTAGCGCTAATTCTCATGTTTGACAGCTTATCCAAAAAGG
4OT34ProNNK_Fwd	CCCTGGATGCGNNKCTGACCAG
4OT34ProNNK_Rev	CAGMNNCGCATCCAGGGAGCGC
4OTGibson_Fwd	GTGAGCGGATAACAATTTCACACAGAATTC
4OTGibson_Rev	ACAGGAGTCCAAGCTCAGCTAATTAAGCTTG

Supplementary References

1 Zandvoort, E., Baas, B.-J., Quax, W. J. & Poelarends, G. J. Systematic screening for catalytic promiscuity in 4-oxalocrotonate tautomerase: enamine formation and aldolase activity. *ChemBioChem* **12,** 602-609 (2011).

2 Waterhouse, A. M., Procter, J. B., Martin, D. M. A., Clamp, M. & Barton, G. J. Jalview Version 2—a multiple sequence alignment editor and analysis workbench. *Bioinformatics* **25,** 1189-1191 (2009).
